# Recent Advances in the Understanding of Vestibular Migraine

**DOI:** 10.1155/2016/1801845

**Published:** 2016-10-16

**Authors:** Jong-Hee Sohn

**Affiliations:** Department of Neurology, Chuncheon Sacred Heart Hospital, Hallym University College of Medicine, Chuncheon-si, Gangwon-do, Republic of Korea

## Abstract

Approximately 1% of the general population and 10% of patients with migraine suffer from vestibular migraine (VM). However, this condition remains relatively unknown; therefore, it is often underdiagnosed despite the recent adoption of international diagnostic criteria for VM. The diagnosis of VM is based on the symptoms, degree, frequency, and duration of the vestibular episodes, a history of migraine, the temporal association of migraine symptoms with vestibular episodes in at least 50% of cases, and the exclusion of other causes. Physical examination and laboratory findings are usually normal in patients with VM but can be used to rule out other vestibular disorders with similar symptoms. The pathophysiology of VM remains incompletely understood; however, several mechanisms link the trigeminal system, which is activated during migraine attacks, and the vestibular system. Because few controlled trials have specifically investigated VM, the treatment options for this order are largely the same as those for migraine and include antiemetics for severe acute attacks, pharmacological migraine prophylaxis, and lifestyle changes.

## 1. Introduction

Since the initial report describing the association between migraine and vertigo [1], a number of studies over the last three decades have shown vestibular migraine (VM) to be a common cause of repeated episodic vertigo. A consensus document published by both the International Bárány Society and the International Headache Society considers VM to be a distinct diagnostic entity. Recently, diagnostic criteria for VM were included in the appendix of the beta version of the International Classification of Headache Disorders-3 (ICHD-3*β*). Therefore, it appears that there is increasing awareness of this emerging entity. As a result, the present review aimed to describe the diagnostic criteria and epidemiology of VM, the current understanding of its pathogenesis, and its treatment options, with a focus on clinical features.

## 2. Diagnostic Criteria for VM

Various terms have been used to describe the relationship between migraine and vestibular symptoms including migrainous vertigo, migraine-associated vertigo or dizziness, migraine-related vestibulopathy, and benign recurrent vertigo [2–6]. The diagnostic criteria for VM were first suggested using the term migrainous vertigo and included recurrent episodic vestibular symptoms of at least moderate severity, a current or previous history of migraine, and migrainous symptoms (migrainous headache, photophobia, phonophobia, and/or visual or other auras) involved in at least two vertiginous attacks [7]. To increase sensitivity and specificity, diagnostic criteria differentiating definite and probable migrainous vertigo were also developed; the validity of these diagnostic criteria was demonstrated in follow-up studies [5, 8]. Subsequently, consensus criteria that were jointly formulated by the Migraine Classification Subcommittee of the International Headache Society and the Committee for Classification of Vestibular Disorders of the Bárány Society were presented [9]. These criteria are stricter than previously suggested diagnostic guidelines and included criteria for both VM and probable VM (see “diagnostic criteria for vestibular migraine”). Diagnostic criteria for VM were also accepted in the appendix of the ICHD-3*β*, which lists new disorders in need of further research for validation [10]. Following the publication of these internationally confirmed diagnostic criteria, more systematic studies of VM will be conducted with the aim of establishing the validity of the diagnostic evaluation of VM in actual clinical situations [11]. The ICHD-3*β* criteria for the diagnosis of VM require that a patient has current or previous migraines with or without aura, a patient has had at least five vestibular episodes of moderate or severe intensity lasting 5 min to 72 h, and at least 50% of these episodes involved 1 or more migrainous symptoms. Although probable VM is not included in the ICHD-3*β*, studies have shown that approximately half of probable VM cases ultimately progress to VM. If future studies support these findings, then probable VM may be included in later versions of the ICHD [9, 12]. 

*Diagnostic Criteria for Vestibular Migraine*

*VM*

- At least 5 episodes fulfilling criteria (C) and (D)
- A current or past history of 1.1 migraine without aura or 1.2 migraine with aura
- Vestibular symptoms of moderate or severe intensity, lasting between 5 min and 72 h
- At least 50% of episodes associated with at least 1 of the following 3 migrainous features
  1. Headache with at least 2 of the following 4 characteristics: unilateral headache, pulsating quality, moderate to severe intensity, or aggravation by routine physical activity
  2. Photophobia and phonophobia
  3. Visual aura
- Not better accounted for by another ICHD-3*β* diagnosis or by another vestibular disorder

*Probable VM*

- At least 5 episodes with vestibular symptoms of moderate or severe intensity, lasting between 5 min and 72 h
- Only 1 of criteria (B) and (D) for VM fulfilled (migraine history or migraine features during the episode)
- Not better accounted for by another ICHD-3*β* diagnosis or by another vestibular disorder

## 3. Epidemiology of VM

The reported prevalence of VM varies according to the different diagnostic criteria and study populations used; its prevalence is reported in the range of 4.3–29.3%, while the prevalence of probable VM is 4–5.7% [4, 5, 13–15]. Prior to the publication of the current diagnostic criteria, the rates of VM were reported to be 4.2–29.3% in otolaryngology clinics, 6–25.1% in specialized dizziness clinics [4, 5, 13–16], and 9–11.9% in headache clinics [5, 17]. Recently, a prospective, first-visit, neurology outpatient-based, multicenter study found the prevalence of VM to be 10.3% in migraine sufferers based on the current ICHD-3*β* criteria and the rate of probable VM to be 2.5% based on the consensus diagnostic criteria agreed upon by the International Headache Society and the Bárány Society [18]. A community-based study conducted in women aged 40–54 years reported that the 1-year prevalence of VM was 5%, which was high [19]. It has lifetime prevalence of VM which was about 1% and 1-year prevalence of 0.9% in the general population [20]. Therefore, VM may be the most common cause of recurrent spontaneous vertigo attacks after benign paroxysmal positional vertigo (BPPV) [21]. Despite the relatively high prevalence of VM, it remains underdiagnosed. This became evident following a report that the diagnosis rate of suspected VM made by referring physicians was 1.8%, whereas the actual VM diagnosis rate was 20.2% when the patients were seen in a tertiary vertigo center [16].

## 4. Clinical Features of VM

VM is 1.5–5 times more frequent in females than in males [2, 4, 5, 22] and it can occur at any time in life; the mean age at first occurrence was 37.7 years for females and 42.4 years for males [2–5]. It has also been proposed that VM has familial clustering that follows an autosomal dominant pattern of inheritance with decreased penetrance in men [23]. In most patients with VM, migrainous headaches begin earlier than vestibular attacks [4, 5]. Previous studies have reported a difference of several years between the onset of migraine and the onset of vestibular episodes in VM cases [12, 24]. In fact, some patient has been free of migraine attacks for years when VM first manifests itself [4]. The association between VM and migraines with aura is still under debate, as some studies have found a connection [1, 4, 5, 25, 26], while others reported that VM occurs more commonly or at least with equal frequency in patients with migraine without aura [4, 22].

Migraine attacks can be replaced by independent vertigo, dizziness, or a transient feeling of disequilibrium in elderly patients, especially postmenopausal women [27]. Additionally, benign paroxysmal vertigo of childhood, as described in the ICHD-II, is considered to be the initial manifestation of VM because many children who suffer from that condition have migraines a few years after the vertigo attacks have ceased [28]. Benign paroxysmal vertigo of childhood is included in the chapter on migraine, among the childhood periodic syndromes that are commonly precursors of migraine, together with cyclic vomiting and abdominal migraine [29].

In VM, rotational or nonrotational vertigo may occur spontaneously or in association with position changes. A large population-based survey conducted using telephone interviews found that the rate of spontaneous rotatory vertigo is 67% while that of positional vertigo is 24% [20]. Furthermore, increased sensitivity to motion, especially movements of the head, has been reported [2, 30]. In a study performed in a headache clinic, the most common symptoms of VM were unsteadiness (91%), balance problems (82%), and lightheadedness (77%) [24], which are not part of the vestibular symptoms included in the current diagnostic criteria of the Bárány Society for VM [31]. Rather, the vestibular symptoms defined as necessary by the Bárány Society to qualify for a diagnosis of VM include spontaneous internal and external spontaneous vertigo, positional vertigo, visually induced vertigo, head motion-induced vertigo, and head motion-induced dizziness with nausea [10]. The most common vestibular symptom in patients with VM is head motion-induced dizziness with nausea (37%) while the most common symptom in patients with probable VM is spontaneous vertigo (44%) [18].

The duration of an attack can vary from a few seconds (10%) to several minutes (30%) or several hours (30%) and even up to a few days (30%) [1, 3, 4, 22]; however, the diagnostic criteria for VM require a minimum duration of 5 min and the attacks rarely exceed 72 h. According to the ICHD, VM cases often do not fulfill the duration criteria for an aura or the temporal relationship of migraine headaches. Only 10–30% of VM patients experience typical vestibular auras with durations of 5–60 min [4, 5] and dizziness or vertigo may proceed the migraine attacks or occur during or after the headache [3, 5, 22]. In fewer than 25% patients, every headache episode is accompanied by dizziness or vertigo [20] but headache does not occur in about 30% of all VM attacks and, in some patients, vertigo and headache do not occur at the same time [3, 5, 22]. Therefore, in these cases, typical migraine-associated symptoms, such as photophobia, phonophobia, osmophobia, nausea, and/or vomiting, are important for diagnosis.

VM has triggering factors that are similar to those of typical migraines, including menstruation, stress, lack of sleep, and diet. Additionally, vertigo induced by vestibular stimulation (rotation/caloric testing) may induce a migraine attack and can act as a specific migraine trigger [32]. Auditory symptoms, such as hearing loss, tinnitus, and ear fullness, are reported in 38% of patients with VM [33] and a long-term follow-up study found that the rate of auditory symptoms accompanying vertigo increased from 15% to 49% over the course of the investigation [34]. Further, the prevalence of ear fullness statistically differs among various VM diagnostic subgroups, such as VM, probable VM, and atypical VM [13]. Hearing loss tends to be transient and mild with or without minor progression during the course of the disease [22] but 18% of VM patients develop mild bilateral sensorineural hearing loss in the low-frequency range [12].

Patients with VM have poor sleep quality, high levels of depression, and a low overall quality of life [20, 35]. Approximately 2 out of 3 patients with VM visit clinicians due to their symptoms; however, VM is diagnosed in only about 20% of these patients [20]. Moreover, slightly different opinions regarding the diagnosis and treatment of VM exist in departments of neurology and otolaryngology. It has been reported that neurologists would diagnose VM in 82% of patients with vertigo and headache, whereas only 64.5% of otolaryngologists would do so; in fact, many specialists (14.5% of neurologists and 19% of otolaryngologists) stated that they had never treated a patient with VM [36]. Therefore, patients may not receive appropriate treatment because VM is often underdiagnosed in actual clinical situations. A detailed medical history and full examination should be conducted for patients who have both migraines and vestibular symptoms, and a clinician must always consider the possibility of VM in these cases.

## 5. Differential Diagnosis for VM

A variety of diseases that may cause episodic vertigo are included in the differential diagnosis of VM (see “differential diagnosis for VM”). In particular, the symptoms of VM may mimic those of Meniere's disease (MD) and BPPV [33]. BPPV should be considered as a differential diagnosis of VM in patients presenting with positional vertigo, and performance of diagnostic provocation maneuvers during an acute episode of vertigo may be the only way to clinically differentiate between these disorders. In VM, attacks of positional vertigo last from a few hours to several days and often occur several times per month or year while BPPV attacks last from several seconds to a few minutes and generally appear over the course of a few weeks or months [12]. Also, the nystagmus also depends on the patient's position but does not match a specific semicircular canal in the case of VM. The primary differential diagnosis is MD. The current diagnostic criteria for both of these disorders are mainly based on the clinical symptoms of a patient because no biological markers are available. However, the signs and symptoms of VM sometimes overlap with those of MD, which may give rise to diagnostic uncertainty [44]. Several practical criteria that can be used to differentiate MD and VM have been suggested [51]. First, reports of only very short (a few seconds to less than 15 min) or prolonged (more than 24 h) vertigo spells are more likely due to VM rather than MD. Additionally, audiometric and vestibular anomalies are typically milder in magnitude and tend to be stable rather than fluctuate over time. Patients with MD primarily suffer mainly from auditory symptoms (tinnitus and hearing loss), while migraine symptoms (migraine headache, photo-/phonophobia, and visual aura), anxiety, and palpitations are more common during attacks of VM [52]. On the other hand, a study that compared patients with VM and MD found that 38% of patients with VM had auditory symptoms, whereas 49% of patients with MD had headaches [33]. Headache compatible with migraine is observed in 20.4% of patients with MD, but the frequency of migraine-type headache is significantly higher in VM than in either probable VM or MD [52]. In the early stages of these disorders, a differential diagnosis between MD and VM may be difficult. Patients with VM may experience all the symptoms of MD, including fluctuating sensorineural hearing loss, even though repeated VM attacks very rarely produce permanent hearing loss [53–55]. Additionally, a retrospective study showed that 13% of patients fulfilled the criteria for both disorders [33]. Due to the similar clinical presentations of these disorders, a follow-up assessment is the only method that can accurately distinguish between VM and MD because most reliable distinguishing features of patients with MD are progressive hearing loss that manifests over several years and low-frequency hearing loss evident on an audiogram [44]. When the criteria for MD are met, particularly hearing loss as evidenced by audiometry, MD should be diagnosed, even when migraine symptoms occur during vestibular attacks. Only patients who have two different types of attacks, one fulling the criteria for VM and the other fulfilling the criteria for MD, should be diagnosed with both disorders. A future revision of the ICHD may include a VM/MD overlap syndrome [10].

*Differential Diagnosis for VM*

1. Meniere's disease (MD)
2. Benign paroxysmal positional vertigo (BPPV)
3. Migraine with brainstem aura
4. Transient ischemia of the vertebrobasilar circulation
5. Central positional vertigo
6. Syncope and orthostatic hypotension
7. Motion sickness
8. Vascular compression or schwannoma of the 8th nerve
9. Episodic ataxia type 2
10. Somatoform vertigo

VM can be diagnosed based on the criteria described in “Diagnostic Criteria for Vestibular Migraine.” This diagnosis should be made after taking a full medical history of the patient that assesses the main vestibular symptoms, the intensity (severity), duration (5 min to 72 h), and frequency (5 times or more) of attacks, history of migraine, time association between migrainous symptoms and vestibular episodes, and the exclusion of other diseases [56]. There are five main vestibular symptoms according to the Bárány Society Classification of Vestibular Symptoms and the qualification criteria for a diagnosis of VM in the appendix of the ICHD-3*β*; a single episode of dizziness is not included in these criteria to increase specificity. The five vestibular symptoms necessary for a diagnosis of VM are spontaneous vertigo, positional vertigo that occurs when changing the position of the head, visually induced vertigo induced by a complex or large moving visual stimulation, head motion-induced vertigo that occurs during head motion, and head motion-induced dizziness with nausea. The duration of an episodes is defined as the total period during which short attacks occur repeatedly during head motion and visual stimulation or after changes of head position [9, 10]. Compared with a usual migraine attack, headache does not often accompany vestibular episodes or is attenuated when migraine occurs in conjunction with vertigo [4]. Therefore, detailed accounting of migrainous symptoms in addition to headache, such as photophobia or auras, is also necessary. Because a diagnosis of VM is made based on medical history and not based on pathognomonic findings from physical examination or laboratory tests, a diagnosis of VM should be considered for patients with a history of migraine when vertigo associated with other migrainous symptoms and other etiologies has been ruled out.

## 6. Physical Examinations and Laboratory Tests

Numerous studies have reported that there are no disease-specific findings indicative of VM on physical examinations or laboratory tests. Additionally, neurotological findings between attacks are usually normal, although abnormalities are found in some patients (see “reports of clinical signs in patients with VM”). On the other hand, mild clinical signs of central vestibular dysfunction, such as gaze-induced nystagmus, central positional nystagmus, and spontaneous nystagmus, have been reported [4]. Furthermore, a follow-up study conducted over 9 years found that interictal ocular motor abnormalities, including positional nystagmus in 12 to 28% of patients and subtle saccadic pursuit in 20 to 63% of patients, increase over time [34]. Peripheral vestibular signs include canal paresis [1–4, 57, 58], mild low-frequency cochlear loss [1, 2, 54], bilateral vestibular failure [1, 2, 58], and mild bilateral sensorineural hearing loss [34]. During attacks, patients with VM can present with pathological spontaneous or positional nystagmus consistent with a peripheral or central vestibular abnormality or a mixture of both peripheral and central features [59]. Additionally, low-velocity sustained nystagmus during positional testing on a symptomatic day is thought to be highly suggestive of VM [60].

*Reports of Clinical Signs in Patients with VM*

*Neurotological Findings*

*Between Attacks*

 Gaze-induced nystagmus (27%) and spontaneous nystagmus (11%) [4]
 Persistent positional nystagmus and positional nystagmus (12→28%) [2, 34]
 Vertical (48%) and/or horizontal (22%) saccadic pursuit [4]
 Subtle saccadic pursuit 20→63% in follow-up study over 9 years [34]
 Unilateral canal paresis (8–22%) [1–4, 57, 58]
 Bilateral vestibular failure (11%) [1, 2, 58]
 Low-frequency, mild cochlear loss (3–12%) [1, 2, 54]
 Mild bilateral sensorineural hearing loss (18%) in follow-up study over 9 years [34]

*During Attacks*

 Spontaneous nystagmus (19% and, nystagmus provoked by horizontal headshaking (35%) [60]
 Low-velocity, sustained, central positional nystagmus (100%) [60]
 Pathologic nystagmus with spontaneous or positional nystagmus (70%) [59]
  - Central vestibular dysfunction (50%)
  - Peripheral vestibular dysfunction (15%)
  - Unclear mixture (35%)

Various abnormalities on laboratory tests, but not pathognomonic findings, have been associated with VM. Approximately 25% of VM cases exhibit a unilateral reduction of peripheral vestibular function and about 50% of VM cases show vestibuloocular asymmetry [61]. Additionally, VM patients have high rates of abnormal cervical or ocular vestibular-evoked myogenic potentials [39, 62] and abnormally high sensitivity to low-frequency dynamic roll tilting [63]. Nonetheless, in clinical practice, the medical history of a patient will usually provide more significant clues leading to a diagnosis of VM than vestibular testing because there are no abnormalities in the latter tests that are specific to VM.

## 7. Pathogenesis of VM

At present, the neural mechanisms of VM remain unknown and require further study; a majority of the current hypotheses are based on knowledge of migraine itself. It has been proposed that the reciprocal connections between brainstem vestibular nuclei and the structures that modulate trigeminal nociceptive inputs may underlie the pathophysiology of VM. Furthermore, different clinical findings from the periods between and during attacks refer to an interaction between the vestibular system and the mechanisms underlying migraine at various levels [64]. Cortical spreading depression, which is thought to give rise to migraine with aura, is considered to be part of the pathogenesis of VM in patients with short-duration vertiginous attacks and may result in vestibular symptoms if it arrives in the vestibular areas of the cortex, which are mainly located in the posterior insula and the temporoparietal junction [3]. However, other clinical symptoms that accompany the acute phase of VM, such as canal paresis or complex positional nystagmus, cannot be explained by cortical dysfunction [65]. Some neurotransmitters involved in the pathogenesis of migraine (e.g., calcitonin gene-related peptide, serotonin, noradrenaline, and dopamine) control the activities of the central and peripheral vestibular nerves, which may be involved in the pathogenesis of VM [2, 3, 22, 64]. Also, serotonin-induced plasma extravasation induced was observed in rats not only in the intradural area but also in the inner ear; a potential peripheral mechanism was reported by this study [66].

VM is related to migraine and exhibits a familial trend [67]. A genetic deficiency in voltage-gated calcium channels was identified in patients with familial hemiplegic migraine and type II episodic ataxia; these two paroxysmal diseases are characterized by vertigo and migraine as the major symptoms [68]. Although it was hypothesized that a genetic deficiency in the same region would be related to VM, this genetic deficiency could not actually be demonstrated [69, 70]. Recently, a variant of ATP1A2 was associated with a new form of progressive hearing loss with migraine in a Korean family [71].

The vestibular and pain pathways are neurochemically similar and may share a central mechanism involved in the perception of sensations [64]. Based on findings from a human experimental model of VM, the only hypothesis that has been proposed thus far involves a reciprocal connection between the trigeminal nervous system and the vestibular nervous system; that is, vestibular nuclei may influence noradrenergic and serotonergic pathways that contribute to the onset of migraine attacks and are involved in modulation of the pain pathway, information processing in the spinal trigeminal nucleus caudalis and thalamocortical mechanisms. Alternatively, it has been proposed that peptide release from the primary vestibulocochlear sensory terminal into inner ear fluids may play a role in VM and that migraine mechanisms may affect vestibular processing via monoaminergic pathways, trigeminovestibular connections, and/or cortical mechanisms [72]. A clinical study reported that spontaneous nystagmus in migraine patients, but not control patients, can be triggered or modulated by painful trigeminal stimulation, which provides evidence of functional connections between the vestibular and trigeminal systems [73].

Recent functional neuroimaging findings have suggested that the dysmodulation of multimodal sensory integration and the processing of vestibular and nociceptive information could be factors related to VM. Positron emission tomography (PET) studies revealed an increase in metabolism in the temporoparietoinsular areas and bilateral thalami during VM attacks, which indicates that there was an activation of the vestibulothalamocortical pathway [74]. Patients with VM also exhibit significant increases in ipsilateral thalamic activation following vestibular stimulation compared with controls and patients with migraine without aura [75]. Moreover, brain regions related to the integration of visual and vestibular cues were activated in VM patients who underwent functional magnetic resonance imaging (MRI) analyses during a visual stimulation procedure in a vertigo-free period [76]. Voxel-based morphometry studies found reductions in gray matter volumes in the inferior temporal gyrus, cingulate cortex, and posterior insula in patients with VM; these areas are involved in the cortical processing of vestibular and nociceptive information [77]. These functional and structural alterations in patients with VM resemble those previously described in patients with migraine; therefore, it is possible that VM represents a pathophysiological connection between migraine and the vestibular system [78].

## 8. Treatment of VM

Treatment for VM is based on expert opinions because few randomized controlled trials have been carried out to identify optimal treatment strategies (Table 1). Two randomized case-controlled trials evaluating the therapeutic effects of triptan during the acute phase of VM have been published [37, 38]: one showed that 38% of VM patients improved when treated with zolmitriptan (5 mg), whereas only 22% of patients in the placebo group improved [37] and the other reported that motion sickness caused by vestibular stimulation in patients with migraine was significantly reduced following treatment with rizatriptan compared with a placebo [38]. Antiemetic medications (e.g., dimenhydrinate and benzodiazepines) may be also useful during the acute phase of VM; methylprednisolone (1,000 mg/day, 1–3 d) has a beneficial impact on patients with VM with continuous severe episodes [79].

Prophylactic medications are mainstays for the management of VM. Because relatively few controlled trials have been conducted to date, most of the drugs used for VM are also used for the prevention of migraine headaches [80]. A review of eight retrospective studies evaluating VM found that preventive medications such as nortriptyline, verapamil, or metoprolol produce marginal clinical improvements [81]. Accordingly, no specific preferred preventive medicines for VM have been established because there is a lack of definite evidence regarding the efficacy of various therapeutic agents. Therefore, the side effects associated with a preventive medication may influence its selection and the effectiveness of preventive medicines should be judged after at least 3 months. Additionally, acetazolamide, dichlorphenamide, and carbonic anhydrase inhibitors are not generally recommended for the preventive treatment of migraines but may be effective for the prevention of VM [82]. Recently, venlafaxine and propranolol show equal effectiveness as prophylactic drugs for ameliorating vertiginous symptoms of VM in prospective, randomized, controlled clinical trial [42]. Another reports do not differ with those of a previous paper, referring to a substantial control of vertigo attacks in subjects with VM by fixed combination of cinnarizine and dimenhydrinate or acetazolamide [48, 49]. Also, an ongoing trial will test the efficacy of metoprolol in VM (prophylactic treatment of vestibular migraine with metoprolol (PROVEMIG) trial) [83].

Nonpharmacological treatment options for VM such as avoiding triggering factors, getting regular sleep and meals, and exercising may also be helpful for VM and should be considered as preventative measures, as with general migraine. Behavioral modifications can be tried. One retrospective study showed that 14% of 38 patients enrolled reported improvement in symptoms after caffeine cessation [43]. Furthermore, vestibular rehabilitation exercises can be helpful, either independently or in conjunction with drug therapy [50].

## 9. Conclusions

VM is a common cause of recurrent episodic vertigo but it is likely underdiagnosed due to the absence of a unified set of diagnostic criteria. However, with the recent addition of new diagnostic criteria in the appendix of the ICHD-3*β*, future studies addressing this issue will aid in the characterization of this disorder. To identify VM, detailed physical examination, the assessment of a patient's medical history regarding vestibular symptoms, and consideration of differential diagnoses to exclude other diseases are necessary. Additionally, in clinical practice, the possibility of VM in patients with a history of migraine or who currently suffer from migraine headaches should always be considered.

## Figures and Tables

**Table 1:** Treatment options for VM.

Treatment options		Clinical trial [reference]
Acute medications		
Zolmitriptan	2.5 mg oral	Randomized controlled trial [37]
Rizatriptan	10 mg oral	Randomized controlled trial [38], motion sickness

Prophylactic medications		
Propranolol	160 mg, 40–160 mg	Retrospective cohort analysis [39–41]
Propranolol/venlafaxine	40~160 mg/37.5~150 mg	Prospective, randomized, controlled clinical trial [42]
Metoprolol	150 mg, 100–200 mg	Retrospective cohort analysis [39, 41]
Amitriptyline	100 mg, 10 mg	Retrospective cohort analysis [39, 41]
Nortriptyline	27–75 mg	Open-label, chart review [43]
Valproic acid	600 mg, 600 mg	Retrospective cohort analysis [44], cohort study, vestibule-ocular reflex [45]
Topiramate	50 mg, 50–100 mg	Retrospective cohort analysis [39], open-label chart review [43]
Lamotrigine	75 mg	Retrospective cohort analysis [39]
Flunarizine	5 mg, 5–10 mg, 5–10 mg	Retrospective cohort analysis [39], retrospective, open-label [41], open-label, postmarketing [46]
Cinnarizine	37.5–75 mg	Retrospective, open-label [47]
Cinnarizine + dimenhydrinate	20 mg and 40 mg	Observational trial [48]
Acetazolamide	500 mg	Retrospective cohort study [49]
Magnesium	400 mg	Retrospective cohort analysis [39]
Clonazepam	0.25–1 mg	Retrospective cohort analysis [41]

Nonmedical treatments		
Vestibular rehabilitation	5 therapy sessions over 9 weeks	Uncontrolled, observational trial [50]
Caffeine cessation	4–6 weeks	Retrospective, observational trial [43]
